# Ad libitum water consumption prevents exercise-associated hyponatremia and protects against dehydration in soldiers performing a 40-km route-march

**DOI:** 10.1186/s40779-019-0192-y

**Published:** 2019-01-25

**Authors:** Heinrich W. Nolte, Kim Nolte, Tamara Hew-Butler

**Affiliations:** 1 Movement Physiology Research Laboratory, School of Physiology, Faculty of Health Sciences, University of the Witwatersrand, Johannesburg, South Africa; 2 Division Biokinetics and Sport Science, Department of Physiology, University of Pretoria, Pretoria, South Africa; 3 Division of Kinesiology, Wayne State University, Detroit, MI USA

**Keywords:** Serum sodium concentration, Exercise-associated hyponatremia, Arginine vasopressin, Fluid balance, Electrolyte balance, Military

## Abstract

**Background:**

It remains unclear if ad libitum water drinking, as a hydration strategy, prevents exercise-associated hyponatremia (EAH) during prolonged exercise. The aim of this study was to determine the incidence of EAH within the broader context of fluid regulation among soldiers performing a 40-km route-march ingesting water ad libitum.

**Methods:**

Twenty-eight healthy male soldiers participated in this observational trial. Pre- and post-exercise body mass, blood and urine samples were collected. Blood samples were assessed for serum sodium ([Na^+^]), glucose, creatinine, urea nitrogen (BUN), plasma osmolality, creatine kinase (CK), and plasma arginine vasopressin (AVP) concentrations. Plasma volume (PV) was calculated using hematocrit and hemoglobin. Urine samples were analyzed for osmolality and [Na^+^]. Water intake was assessed by weighing bottles before, during and after the march. The mean relative humidity was 55.7% (21.9–94.3%) and the mean dry bulb temperature was 27.1 °C (19.5 °C - 37.0 °C) during the exercise.

**Results:**

Twenty-five soldiers (72 ± 10 kg) (Mean ± SD) completed the march in 09:11 ± 00:43 (hr:min). Participants consumed 736 ± 259 ml/h of water and lost 2.8 ± 0.9 kg (4.0% ± 1.4%, *P* < 0.05) of body mass. Significant (pre-march vs. post-march; *P* < 0.05) decreases in serum [Na^+^] (141 mmol/L vs. 136 mmol/L), plasma osmolality (303 mOsmol/kg H_2_O vs. 298 mOsmol/kg H_2_O), and serum creatinine (111 μmol/L vs. 101 μmol/L) and urine [Na^+^] (168 mmol/L vs. 142 mmol/L), as well as significant increases in plasma AVP (2 pg/ml vs. 11 pg/ml), plasma CK (1423 U/L vs. 3894 U/L) and urine osmolality (1035 mOsmol/kg H_2_O vs. 1097 mOsmol/kg H_2_O) were found. The soldier (72 kg) with the lowest post-exercise sodium level completed the march in 08:38. He drank 800 ml/h, lost 2% body mass, and demonstrated (pre-post) increases in plasma osmolality (294–314 mOsmol/kg H_2_O), BUN (20–30 mg/dl), AVP (2–16 pg/ml) and PV (41%). His urine osmolality decreased from 1114 mOsmol/kg H_2_O to 1110 mOsmol/kg H_2_O. No participants finished the route-march with a serum [Na^+^] indicating hypernatremia (range, 134–143 mmol/L).

**Conclusions:**

Ad libitum drinking resulted in 4% body mass loss with a 2 mmol/L serum [Na^+^] reduction in conjunction with high urine osmolality (> 1000 mOsmol/kg H_2_O) and plasma AVP. No single hydration strategy likely prevents EAH, but hypernatremia (cellular dehydration) was not seen despite > 2% body mass losses and high urine osmolality.

## Background

Exercise-associated hyponatremia (EAH) is a potentially fatal sodium imbalance (serum sodium concentration < 135 mmol/L) resulting from a loss of solutes (sodium and potassium), relative excess of total body water, or likely a combination of both [1]. While all three mechanisms are plausible in the development of low serum sodium concentrations ([Na^+^]), evidence suggests that total body water expansion relative to the amount of total body exchangeable sodium (Na^+^) is the main pathogenic cause of both asymptomatic and symptomatic EAH [2]. The under-replacement of sweat sodium losses appears to be of greater pathogenic significance during long, hot events, particularly in individuals with elevated sweat sodium losses [3–5].

Drinking according to thirst is widely advocated as the most appropriate individualized hydration strategy to prevent both hyponatremia and hypernatremia (serum [Na^+^] > 145 mmol/L; or cellular dehydration) [5, 6]. However, persisting debates about hypovolemic-driven thirst [4, 7], conceptual confusion over ad libitum versus thirst-mediated drinking [8], and concerns about the deleterious effects of dehydration on performance [9] often question the efficacy of drinking to thirst as a valid hydration strategy. Most hydration strategies advocated by sports medicine specialists aim to minimize the deleterious health and performance effects of dehydration [10]. As such, most of the current hydration guidelines advocate that athletes drink above thirst to limit body mass losses to < 2% and/or maintain a dilute urine output (urine osmolality < 700 mOsmol/kg H_2_O), with sodium ingestion as the main strategy to prevent EAH during exercise [10].

Due to a prior tragedy involving a soldier who died from complications due to both EAH and exertional heatstroke during a 50-km march [11], we wished to critically examine fluid balance and natremia status in soldiers only given access to water during a long duration march in a temperate climate. Therefore, the aim of the study was to determine the incidence of EAH among a group of South African National Defence Force (SANDF) soldiers performing a 40-km route-march while drinking only water (no food or sodium) ad libitum. Our hypothesis was that normonatremia would be maintained in soldiers drinking water ad libitum.

## Methods

### Ethics committee approval and informed consent

Submission was made to and approval given by the South African Military Health Service (SAMHS) 1 Military Hospital Research Ethics Committee for the study. Soldiers taking part in a selection program were recruited as possible participants. All participants were briefed and asked to voluntarily sign an informed consent form prior to commencing with the trial.

### Route-march, water intake and body mass change

The study utilized a route-march covering 40 km that was part of a selection preparation program. The march was time restricted, and participants were instructed to complete it as fast as possible. Fluid intake was limited to water ad libitum (as desired). Ad libitum drinking was explained to participants as drinking whenever and whatever volume they desired along the route [9]. The route made use of a combination of graded dirt road and sand and was fairly level. The mass of equipment that participants carried was 55 kg and included, but was not limited to, bush hat/cap, personal battle-jacket, 8 L of water, personal weapon and sand bags in a rucksack to make up the balance of the required payload. Soldiers wore a standard issue battledress clothing configuration. In addition to the water carried as part of the payload, it was communicated to the participants that water would be available for replenishment at 5-km intervals as provided by water bunkers along the route. First line medical support was present during the entire duration of the march. The Wet Bulb Globe Temperature (WBGT) index, dry bulb temperature and relative humidity were monitored at one-minute intervals throughout the duration of the route-march. Participants were weighed (to the nearest 0.02 kg) 1 h before and within 30 min after the route-march wearing only their underclothing. At completion of the march, each participant was provided with a towel to dry excess perspiration prior to re-weighing. Stature was measured to the nearest millimeter with a Harpenden stadiometer (Holtain Limited, UK), 1 h prior to the start of the march (barefoot and in the Frankfort plane). All water bottles were weighed before, during (to account for re-filling along the route) and on completion of the march to determine water consumption during the exercise period. The time it took for each individual to complete the route-march was recorded.

### Hematology and urine variables

Before and after completion of the route-march, a blood sample (5 ml) was collected from the antecubital vein with the participant in a seated position. Participants were seated for 30 min prior to the pre-exercise sample and were allowed a 30-min rest and cool down period after completion of the march before the second sample was drawn. No fluids were allowed during this period. This period allowed for the return of exercise-induced plasma volume (PV) shifts to pre-exercise levels. The blood samples were analyzed on-site for concentrations of serum [Na^+^], potassium ([K^+^]), chloride ([Cl^−^]), ionized calcium ([iCa^++^]) blood glucose, creatinine, hemoglobin (Hb), hematocrit (Hct) and urea nitrogen (BUN) using an I-Stat™ portable analyzer (Abbott, USA). An independent laboratory performed analyses to determine plasma osmolality (POsm), plasma creatine kinase (CK), and plasma arginine vasopressin concentrations ([AVP]_p_). Blood samples used for [AVP]_p_ determination were immediately placed on ice and centrifuged within 10 min at 3000 rpm, separated plasma was frozen, and plasma remained frozen throughout transport to the laboratory. Changes in PV were determined from the pre- and post-exercise Hb and Hct values using the equations of Dill and Costill [12]. Glomerular-filtration rate (GFR) was estimated (eGFR) using the Cockroft-Gault equation [13]. Before and after completion of the route-march, participants emptied their bladders and provided urine samples for analysis of osmolality (UOsm) and U[Na^+^].

### Statistical analysis

To determine which statistical test would be most suited for the comparisons of pre- and post-exercise values, the differences (paired differences) between the results were calculated. The distributions (in the form of histograms) of the paired differences of all the results were plotted with the number of classes calculated according to the Rule of Sturge. The normality of this distribution was tested by means of the Shapiro-Wilks *W*-test. A Pearson’s product–moment correlation coefficient was used to determine the relationships between appropriate variables. Unpaired t-tests were used to perform a sub-analysis to compare soldiers whose serum [Na^+^] declined the most (≥5 mmol/L) and ended the march close to the biochemical cut-off value for EAH versus those soldiers who better maintained normonatremia during the march with ad libitum replacement. Statistical significance was set at *P* < 0.05. The statistical analyses were completed using the Statistical software package.

## Results

### Participants and environmental conditions

Twenty-eight male volunteer soldiers were recruited, with 25 completing the route-march with a full dataset (pre- and post-march). On average, the participants were 26 years old (range, 21–30 years) and had a mean stature of 1759 mm (range, 1632–1942 mm). The WBGT index, dry bulb temperature and relative humidity were monitored from the start of the route-march (03:00) until 13:30 when the last participant completed the exercise. The mean WBGT index during the route-march was 24.0 °C (18.7–30.0 °C), mean relative humidity was 55.7% (21.9–94.3%), and the mean dry bulb temperature was 27.1 °C (19.5–37.0 °C).

### Sub-analysis of sodium serum change during route-march

Five soldiers demonstrated a decrease in serum [Na^+^] equal to or greater than 5 mmol/L over the route-march and lost significantly less body mass compared with those soldiers (*n* = 20) whose serum [Na^+^] remained relatively stable (serum [Na^+^] decrease − 5.4 mmol/L vs. -1.6 mmol/L; *P* < 0.0001, Table 1).

**Table 1:** Body mass change, water intake and route-march time [(Mean ± SD (min – max)]

Item	Combined (*n* = 25)	Serum [Na^+^]	
		≥ 5 mmol/L Δ (*n* = 5*)*	< 5 mmol/L Δ (*n* = 20)
Body Mass Pre (kg)	73.73 ± 9.70 (60.32–98.76)	73.96 ± 5.50 (68.82–81.36)	73.68 ± 9.70 (60.32–98.76)
Body Mass Δ (%)	−4.0 ± 1.4 (− 0.6 – − 7.2)^**^	−3.0 ± 1.7 (− 0.6 - -4.5)^*^	−4.4 ± 1.2 (− 2.7 - -7.2)
Total Water Intake (L)	6.7 ± 2.3 (2.9–11.8)	7.4 ± 1.7 (5.9–9.8)	6.3 ± 2.3 (2.9–11.8)
Hourly Water Intake (ml/h)	736 ± 259 (290–1245)	853 ± 198 (684–1166)	691 ± 270 (290–1245)
Route-March Time (hh:mm)	09:11 ± 00:42 (08:19–10:25)	8.72 ± 0.29 (8.19–9.17)	9.26 ± 0.69 (8.40–10.25)

^*^P < 0.05 between plasma [Na^+^] ≥ 5 mmol/L Δ vs. < 5 mmol/L Δ; ^**^P < 0.05 between pre- and post-route-march values

A single soldier (4%) finished the march with a serum [Na^+^] marginally below (134 mmol/L) the biochemical threshold for hyponatremia (< 135 mmol/L), without clinical signs or symptoms of EAH. This soldier lost the second least amount of body mass (1.42 kg) during the march. He demonstrated the largest decrease in serum [Na^+^] during the march (6 mmol/L) and had the lowest pre- and post-march urine [Na^+^] compared with the rest of the normonatremic cohort (pre-urine [Na^+^]: 61 mmol/L vs. 173 mmol/L and post urine [Na^+^]: 50 mmol/L vs. 150 mmol/L; EAH case vs. normonatremic cohort, respectively). This hyponatremic soldier also demonstrated a pre- to post-march increase in plasma volume (41% vs. -14%) and plasma osmolality (20 mOsmol/kg H_2_O vs. -5 mOsmol/kg H_2_O) when compared with the normonatremic cohort.

### Body mass change, water intake and route-march time

Table 1 presents the body mass change, water intake and route-march time in soldiers as a total group (combined; *n* = 25) and then divided into soldiers who demonstrated a ≥ 5 mmol/L decrease (*n* = 5) versus a <  5 mmol/L decrease (*n* = 20) in serum [Na^+^] post-march minus pre-march (Δ).

### Blood and urine variables

Table 2 presents serum (S), plasma (P) and urine (U) variables in soldiers as a total group (combined; *n* = 25) and then divided into soldiers who demonstrated a ≥ 5 mmol/L decrease (*n* = 5) versus a <  5 mmol/L decrease (*n* = 20) in serum sodium concentration ([Na^+^]) post-march minus pre-march (Δ).

**Table 2:** Serum (S), plasma (P) and urine (U) variables measured pre- and post-march [(Mean ± SD (min – max)]

Item	Combined (*n* = 25)	Serum [Na^+^]	
		≥ 5 mmol/L Δ (*n* = 5*)*	< 5 mmol/L Δ (*n* = 20)
S [Na^+^] Pre (mmol/L)	141 ± 1 (140–143)	141 ± 1 (140–143)	141 ± 1 (140–143)
S [Na^+^] Post (mmol/L)	139 ± 2 (134–143)^@^	135 ± 1 (134–138)^***^	140 ± 2 (138–143)
S [Cl^−^] Pre (mmol/L)	105 ± 2 (100–109)	104 ± 2 (101–107)	105 ± 2 (100–109)
S [Cl^−^] Post (mmol/L)	105 ± 2 (100–110)	102 ± 2 (100–104)^**^	106 ± 2 (102–110)
P Osm Pre (mOsmol/kg H_2_O)	303 ± 8 (294–320)	300 ± 5 (294–307)	303 ± 8 (294–320)
P Osm Post (mOsmol/kg H_2_O)	298 ± 8 (285–323)^@^	297 ± 11 (285–314)	299 ± 8 (289–323)
P [AVP] Pre (pg/ml)	2.2 ± 1.5 (0.5–7.8)	1.4 ± 0.6 (0.5–2.2)	2.4 ± 1.6 (0.5–7.8)
P [AVP] Post (pg/ml)	11.2 ± 6.4 (1.8–32.8)^@^	11.4 ± 3.5 (6.8–14.3)	11.5 ± 7.1 (3–32.8)
S [BUN] Pre (mg/dl)	22.4 ± 4.4 (12.6–32.7)	21.6 ± 3.9 (16.2–26.3)	22.9 ± 3.6 (12.6–32.7)
S [BUN] Post (mg/dl)	28.9 ± 4.0 (21.8–35.0)^@^	30.9 ± 5.5 (21.8–35.0)	28.5 ± 5.5 (22.1–34.7)
S [Glucose] Pre (mmol/L)	4.9 ± 0.7 (3.5–6.1)	5.4 ± 0.5 (4.8–6.1)	4.8 ± 0.8 (3.5–6.1)
S [Glucose] Post (mmol/L)	4.8 ± 1.0 (3.1–7.7)	4.0 ± 0.5 (3.1–4.5)^*^	5.1 ± 1.1 (4.0–7.7)
S [CK] Pre (U/L)	1422 ± 830 (426–3192)	1148 ± 521 (530–1652)	1346 ± 760 (426–3192)
S [CK] Post (U/L)	3893 ± 6359) (933–5121)	2301 ± 543 (1604–2860)	2696 ± 1135 (923–5121)
S [Creatinine] Post (ʮmol/L)	111 ± 18 (73–155)	107 ± 12 (93–125)	114 ± 18 (73–155)
S [Creatinine] Post (ʮmol/L)	100 ± 15) (63–137)	105 ± 14 (83–125)	98 ± 14 (63–137)
P volume Δ (%)	−11.9 ± 25.1 (− 27.3–27.5)	−4.7 ± 32.9 (− 19.7–24.8)	−13.7 ± 23.4 (− 27.3–27.5)
U [Na^+^] Pre (mmol/L)	168 ± 37 (61–230)	141 ± 58 (61–221)	175 ± 31 (100–230)
U [Na^+^] Post (mmol/L)	142 ± 54 (50–257)^@^	95 ± 32 (50–141)^*^	159 ± 53 (56–257)
U Osm Pre (mOsmol/kg H_2_O)	1025 ± 156 (747–1362)	1002 ± 185 (747–1231)	1045 ± 139 (795–1362)
U Osm Post (mOsmol/kg H_2_O)	1097 ± 136 (788–1388)^@^	1046 ± 198 (788–1313)	1114 ± 125 (845–1388)

^*^P < 0.05; ^**^P < 0.01; ^***^P < 0.001; between plasma [Na^+^] ≥ 5 mmol/L Δ vs. < 5 mmol/L Δ; ^@^*P* < 0.05 between pre-march vs. post-march

*Na*^+^ Sodium, *Cl*^−^ Chloride, *Osm* Osmolality, *AVP* Arginine vasopressin, *BUN* Blood urea nitrogen, *CK* Creatine kinase

### Relationships between serum and urine sodium change variables

The change (post-march minus pre-march; Δ) in serum [Na^+^] (mmol/L) was significantly (*P* < 0.05) negatively correlated with % body mass Δ (*r* = − 0.50), total water consumption (*r* = − 0.46), water consumed per hour (*r* = − 0.49), serum [K^+^] Δ (*r* = − 0.52), and [BUN] Δ (*r* = − 0.40). Serum [Na^+^] Δ was significantly positively correlated with post-march serum [Cl^−^] (*r* = 0.79), serum [Cl^−^] Δ (*r* = 0.64), post-march serum [iCa^++^] (*r* = 0.44), POsm (*r* = 0.41), pre-march urine [Na^+^] (*r* = 0.42), and post-march urine [Na^+^] (*r* = 0.46).

The change (post-march minus pre-march; Δ) in urine [Na^+^] was significantly negatively related to blood creatinine concentration Δ (*r* = − 0.45) and plasma [CK] Δ (*r* = − 0.45), while it was significantly positively related to % eGFR Δ (*r* = 0.45) and %PV Δ (*r* = 0.47). Of particular note, pre-march UOsm was significantly (*P* < 0.05) positively related to pre-march serum [Na^+^] (*r* = 0.44), [BUN] (*r* = 0.45), and [AVP]_p_ (*r* = 0.45), while post-march [AVP]_p_ was negatively associated with post-march glucose (*r* = − 0.73). The change in body mass during exercise was significantly negatively related to post-exercise serum [Na^+^] (*P* < 0.05; *r* = − 0.55).

## Discussion

One soldier, drinking water ad libitum, finished the 40-km route-march with asymptomatic biochemical hyponatremia. The degree of EAH in this participant was mild (post-march serum [Na^+^] of 134 mmol/L, which is just below the 135 mmol/L threshold that biochemically distinguishes hyponatremia from normonatremia). Whether or not this soldier drank according to the physiological dictates of thirst could not be determined because thirst rating was not assessed. However, a study performed on endurance cyclists suggested that ad libitum fluid intake volumes were similar to those cyclists who drank when thirsty [8]. We therefore deem these two concepts as similar for the purpose of our discussion. However, evaluating his consumption for the entire march per 10-km intervals could confirm that he did not drink according to a predetermined “schedule” of a set volume per time/distance interval, as is the case among many militaries in the world, however, but a practice that is discouraged within the SANDF.

The mechanism contributing to below normal serum [Na^+^] levels in this soldier appears to be mixed, with contributions from both abnormal water retention and under-replaced sodium losses. The presence of water retention was evidenced by a non-suppressed post-march [AVP]_p_ level (14.3 pg/ml vs. 11.3 pg/ml), reduced eGFR (80 ml/min vs. 104 ml/min) and body mass loss that was less than that of the normonatremic cohort (2% vs. 4%; EAH case vs. normonatremic cohort, respectively). Under-replaced sweat sodium losses may have played a minor role in the development of EAH because this soldier had the lowest pre-march (61 mmol/L) and post-march (50 mmol/L) urine [Na^+^] of all soldiers tested. This low urine [Na^+^] indicates that he was conserving urinary sodium through activation of aldosterone from a low sodium diet and/or high sweat [Na^+^], neither of which were assessed in this study. This hyponatremic soldier also demonstrated an increase in [BUN], POsm and plasma volume. The most plausible explanation for these atypical findings (in association with hyponatremia) suggests that EAH may have resulted from an uncommon presentation of pseudohyponatremia due to increased urea levels within the vascular space. A pseudohyponatremia induced by an elevated urea concentration is possible; however, it is generally considered an uncommon phenomenon because urea is not an effective osmole (like glucose) [14].

Because of the difficulty interpreting one individual’s data EAH against the other (normonatremic) soldiers, we subdivided the data into two groups based on the level of serum sodium decline pre-march to post-march (≥5 mmol/L vs. < 5 mmol/L decrease in serum [Na^+^]). This division of data revealed that the greater decrease in serum [Na^+^] was due to increased water intake (Table 1). This interpretation is supported by significantly lower serum [Cl^−^], glucose, and urine [Na^+^] in the ≥5 mmol/L serum [Na^+^] decrease group, which are all suggestive of dilution over sodium conservation (Table 2) [5]. Whether or not the heightened ad libitum water intake was due to a polymorphism within either the 5-HHT and/or BDKBR2 gene [15] or because of other factors such as xerostomia [16] or a need to drink beyond thirst remains unclear [8].

Most hydration guidelines recommend that athletes drink beyond thirst to prevent the detrimental health and performance consequences of dehydration [10]. Alternatively, although it is suggested that sodium intake during exercise will prevent hyponatremia [10], some studies support this claim [17], while other do not [5]. Accordingly, recommendations to drink enough fluids to maintain body mass losses < 2% or keep urine clear (UOsm < 700 mOsmol/kg H_2_O) [10] would have likely induced hyponatremia instead of preventing cellular dehydration (hypernatremia), which did not occur in this cohort with ad libitum drinking. With mean body mass losses hovering around 4%, UOsm levels above 1000 mOsmol/kg H_2_O both pre-march and post-march, and an overall decline in serum [Na^+^], water intake above ad libitum would have certainly increased the incidence and severity of EAH in these soldiers.

The overall maintenance of normonatremia in these soldiers, with access to only water (no food or supplements) over 09:11 (hh:mm) of continuous marching, is consistent with previous military studies with a few notable exceptions. Recent data published by the United States Armed Forces Health Surveillance Center [18] indicate 1506 (incidence rate of 6.6 per 100,000 person-years [p-yrs]) incidents of EAH among active component members between 1999 and 2014. The highest incidence rate was documented in 2010 (12.6 per 100,000 p-yrs), which resulted in erroneous US military fluid replacement guidelines being revisited to limit the development of further cases. This amendment led to a more than 50% decrease in the incidence rate (5.3 per 100,000 p-yrs) in 2013, before there was what appeared to be another increase in the incidence of EAH (7.1 per 100,000 p-yrs) in 2014.

Similar data are not available for the SANDF. Available data are limited to research participants and are not a true indication of the incidence among SANDF soldiers during either training and/or operational conditions. Since 2009, various hydration-related research projects have been conducted for which 169 soldiers volunteered to participate [19–21]. During this time, 2 soldiers (1.1%) developed EAH (based on serum [Na^+^] assessments) according to the internationally accepted clinical definition of EAH: a serum [Na^+^] below 135 mmol/L [6]. Both cases (one during the current study and another that was a fatal case due to combined exercise-associated hyponatremic encephalopathy and exertional heatstroke) developed during exercises over distances of 40 and 50 km, respectively [11]. Recently, three cases of US soldiers suffering from EAH associated with exertional heat illness were reported [22]. No cases were recorded during previous SANDF research projects that assessed performance over shorter exercise distances (ranging from 16.4 to 25.0 km). This is not an unexpected finding as exercise duration in excess of 4 h is considered one of the key risk factors of developing EAH.

Lastly, from a fluid regulatory perspective, the origins of the exercise-induced water retention verified in this cohort remain unclear. Non-osmotic AVP secretion remains the main pathogenic factor in the development of EAH [5], but the exact stimulus remains elusive. Nausea/vomiting, heat, stress, inflammatory markers (i.e., interleukin-6), hypoglycemia and hypovolemia remain at the forefront of potential exercise-induced non-osmotic stimuli [5]. Several studies have demonstrated increases in AVP after induction of hypoglycemia [14]. Recently, the data of Cairns and Hew-Butler [23] could not verify these findings, although their data did show a positive correlation approaching significance between [AVP]_p_ and blood glucose levels in a hyponatremic subgroup [13]. Our results did, however, demonstrate a significant and strong relationship between [AVP]_p_ and blood glucose levels, supporting the possible contribution of hypoglycemia to the non-osmotic stimulation of AVP. It is acknowledged that continuous exercise, especially of such a strenuous nature, is not routinely performed with access to only water unless due to the consequences of soldier deployments or extreme training and/or selection programs. Most commonly, soldiers would have access to some form of nutrition, which would contribute to Na^+^ balance. However, the practical significance for military training remains that care should be taken during prolonged periods of poor nutrition combined with prolonged exercise, as this may not only influence performance but also possibly fluid balance. Of additional significance, pre-march UOsm was significantly correlated with both pre-march [AVP]_p_ and serum [Na^+^], which suggests appropriate osmotic regulation of AVP at rest but not during exercise.

A limitation of this study was that neither sweat [Na^+^] nor urine volumes were measured. Additionally, serial measures of the variables investigated, as opposed to only pre- and post-measures, may have provided greater insight into fluid balance regulation of the soldiers. However, this was not possible due to the competitive nature of the exercise.

## Conclusions

Ad libitum drinking overall resulted in an 4% body mass loss with an 2 mmol/L serum [Na^+^] reduction in conjunction with high urine osmolality (> 1000 mOsmol/kg H_2_O) and AVP. No single hydration strategy likely prevents EAH, but hypernatremia (cellular dehydration) was not seen despite > 2% body mass losses and high urine osmolality. This is not a surprising finding as it has previously been shown that the change in body mass is a poor surrogate for changes in total body water during prolonged exercise [14, 19, 20, 24, 25]. Water intake in excess of ad libitum volumes would most likely have increased the incidence of EAH in these soldiers. This confirms that drinking according to thirst should be advocated as the most appropriate individualized hydration strategy to prevent both hyponatremia and hypernatremia during prolonged exercise in temperate environments.

## Abbreviations

[AVP]_p_: Plasma arginine vasopressin concentration
ADH: Anti-diuretic hormone
AVP: Arginine vasopressin
BUN: Blood urea nitrogen
BV: Blood volume
CK: Creatine kinase
CV: Cell volume
EABV: Effective arterial blood volume
EAH: Exercise-associated hyponatremia
eGFR: Estimated glomerular filtration rate
GFR: Glomerular filtration rate
Hb: Hemoglobin
Hct: Hematocrit
IL-6: Interleukin-6
POsm: Plasma osmolality
PV: Plasma volume
SANDF: South African National Defence Force
Serum [Na^+^]: Serum sodium concentration
SIADH: Syndrome of inappropriate anti-diuretic hormone secretion
U[Na^+^]: Urine sodium concentration
UOsm: Urine osmolality
WBGT: Wet bulb globe temperature
